# Unhealthy Bread

**Published:** 1885-05

**Authors:** 


### Unhealthy Bread.

English bakers use large quantities of alum in their bread to make it white, moist and soft. If used too freely or too long, alum, like other astringents, deranges the whole machinery of the body. Leibig, the great German chemist, has found, that if bread is made of water, saturated with lime, white-wash may do, it has all the effects of making bread white, soft and moist, without the injurious results of alum. Our own opinion is, that bread made out of wheaten flour is good enough for ordinary people and purposes, without adding powdered rocks to it. We know of no authority for feeding people on rocks, or for supposing that the essence of rock has any nutriment in it. Strong and numerous facts seem to warrant the opinion, that people who drink limestone water, are more liable to cholera, and we have no reason to imagine that mixing flour with the limestone water, makes any organic change in the lime; we may rather sa ely infer, that eating lime, is not any more healthful than drinking lime. Alum is quite heavy enough, without putting the bakers up to the trick of putting rocks in their bread. Selling stones at six cents a pound would be a profitable business. We recommend our readers to use the old-fashioned bread made of flour, with milk and common "rising," and let the Dutch revel in rock bread and saurkraut to their heart's content.

